# Importin α5 negatively regulates importin β1-mediated nuclear import of Newcastle disease virus matrix protein and viral replication and pathogenicity in chicken fibroblasts

**DOI:** 10.1080/21505594.2018.1449507

**Published:** 2018-04-24

**Authors:** Zhiqiang Duan, Haixu Xu, Xinqin Ji, Jiafu Zhao, Houqiang Xu, Yan Hu, Shanshan Deng, Shunlin Hu, Xiufan Liu

**Affiliations:** a Key Laboratory of Animal Genetics, Breeding and Reproduction in The Plateau Mountainous Region, Ministry of Education, Guizhou University, Guiyang, China; b College of Animal Science, Guizhou University, Guiyang, China; c Key Laboratory of Animal Infectious Diseases of Ministry of Agriculture, Yangzhou University, Yangzhou, China

**Keywords:** Newcastle disease virus, matrix protein, nuclear localization signal, nuclear import mechanism, chicken fibroblasts

## Abstract

The matrix (M) protein of Newcastle disease virus (NDV) is demonstrated to localize in the nucleus via intrinsic nuclear localization signal (NLS), but cellular proteins involved in the nuclear import of NDV M protein and the role of M's nuclear localization in the replication and pathogenicity of NDV remain unclear. In this study, importin β1 was screened to interact with NDV M protein by yeast two-hybrid screening. This interaction was subsequently confirmed by co-immunoprecipitation and pull-down assays. *In vitro* binding studies indicated that the NLS region of M protein and the amino acids 336–433 of importin β1 that belonged to the RanGTP binding region were important for binding. Importantly, a recombinant virus with M/NLS mutation resulted in a pathotype change of NDV and attenuated viral replication and pathogenicity in chicken fibroblasts and SPF chickens. In agreement with the binding data, nuclear import of NDV M protein in digitonin-permeabilized HeLa cells required both importin β1 and RanGTP. Interestingly, importin α5 was verified to interact with M protein through binding importin β1. However, importin β1 or importin α5 depletion by siRNA resulted in different results, which showed the obviously cytoplasmic or nuclear accumulation of M protein and the remarkably decreased or increased replication ability and pathogenicity of NDV in chicken fibroblasts, respectively. Our findings therefore demonstrate for the first time the nuclear import mechanism of NDV M protein and the negative regulation role of importin α5 in importin β1-mediated nuclear import of M protein and the replication and pathogenicity of a paramyxovirus.

## Introduction

Newcastle disease virus (NDV), a member of the genus *Avulavirus* in the family *Paramyxoviridae*, is an important avian pathogen that causes substantial economic losses to the poultry industry worldwide [1,2]. The genome of NDV is a non-segmented, single-stranded, negative-sense RNA encoding at least six proteins in the order 3'-NP-P-M-F-HN-L-5' [3]. Of all these viral structural proteins, the matrix (M) protein has the least molecular weight of around 40 kDa and forms an outer protein shell around the nucleocapsid, which constitutes the bridge between the viral envelope and the nucleocapsid [4]. Like most paramyxovirus M proteins, the NDV M protein is a multifunctional nucleocytoplasmic shuttling protein and plays crucial roles in NDV life cycle [5]. In addition to functioning for the assembly and budding of progeny virions in the cytoplasm and at the cell membrane later in infection [6], the NDV M protein is localized in the nucleus and nucleolus early in infection and remains in the nucleoli throughout infection [7–9]. The nuclear-nucleolar localization of NDV M protein is thought to inhibit host cell transcription and protein synthesis similar to the human respiratory syncytial virus (HRSV) [10] and vesicular stomatitis virus (VSV) M protein [11], and also ensure that viral replication and transcription in the cytoplasm proceed smoothly, which is by analogy with the measles virus (MeV) M protein [12]. Numerous studies have demonstrated that the nuclear and nucleolar localization of viral proteins depend on their own nuclear localization signal (NLS) and nucleolar localization signal (NoLS) as well as the cellular transport proteins [13–15]. Our recent study found that the nucleolar protein B23 targets NDV M protein to the nucleoli by interacting with the amino acids 30–60 (a putative NoLS) of M protein and enhances viral replication ability [16]. Previous study has shown that NDV M protein enters the nucleus via a bipartite NLS (KKGKKVIFDKIEEKIRR) independent of other viral proteins [17], but cellular proteins involved in the nuclear import of NDV M protein and the biological functions of this nuclear localization still remain unknown.

It has been demonstrated that nuclear transport of proteins carrying NLS through the nuclear envelope-embedded nuclear pore complexes (NPCs) is mediated by members of the importin superfamily including importin α and importin β [18–20]. The classical paradigm for nuclear import pathway is that importin α directly recognizes and binds to the NLS of cargo proteins, and then importin β1 directs binding of the binary complex to the cytoplasmic side of the NPC. The translocation of GDP-bound small GTPase Ran (RanGDP) conjunct ternary complex through the NPC is mediated by NTF2 via interaction with nucleoporins. Once inside the nucleus, binding of GTP-bound small GTPase Ran (RanGTP) to importin β1 causes the dissociation of the ternary complex [21,22]. Thus, cargo proteins are transported into the nucleus. In general, classical NLSs including monopartite and bipartite NLSs are imported by importin α/β1 heterodimer, while non-classical NLSs can be more complex in length, sequence and amino acid composition that are imported by importin β1 [21]. However, recent studies have found that classical NLSs can also be recognized and binded by importin β1 or homologs without the participation of importin α [23–25]. Moreover, some studies even confirmed that importin α can act as negative regulators for the nuclear import of some cargo proteins mediated by importin β1 alone [26,27]. Therefore, such diverse nuclear import pathways are receiving increasingly attention.

Many recent studies have proven that nuclear localization of viral proteins is crucial for viral replication and propagation [28–31]. For example, nuclear localization of the nucleocapsid protein of porcine respiratory and reproductive syndrome virus is essential for optimal virus replication and inhibition of cellular antiviral processes [32], and the successful production of infectious virions and efficient propagation of Japanese encephalitis virus require the nuclear localization of core protein [33,34]. For the members of paramyxoviruses, the M proteins of HRSV, Sendai virus (SeV), Nipah virus (NiV), and NDV all have the molecular mass less than 40 kDa and localize in the nucleus during the course of virus infection [5]. Although functional NLSs in the M proteins of these viruses have been characterized, so far, only the HRSV M protein is demonstrated to be recruited into the nucleus through direct recognition by importin β1 [35]. In addition, nuclear localization of M protein is important for the generation of HRSV progeny virions and is associated with the pathogenesis of viral infection [36]. Therefore, we speculated that the cellular importin members might also participate in the nuclear import of NDV M protein and regulate the replication and pathogenicity of NDV.

In this study, importin β1 was identified to be the nuclear transport receptor of NDV M protein and mediate the nuclear import of NDV M protein by binding its NLS region via the RanGTP-dependent pathway. Further studies showed that NLS mutation in the M protein disrupted its nuclear localization and reduced viral replication in chicken fibroblasts and attenuated viral replication and pathogenicity in SPF chickens. Interestingly, importin α5 was demonstrated to be a negative regulator in importin β1-mediated nuclear import of M protein and the replication and pathogenicity of NDV, as importin α5 deletion remarkably increased the nuclear accumulation of M protein and the replication ability and pathogenicity of NDV in chicken fibroblasts. Our studies will provide deep insights into understanding the more functions of M's nuclear localization in the life cycle and pathogenesis of NDV.

## Results

### Yeast two-hybrid screening of NDV M-interacting cellular proteins

**Figure 1.**
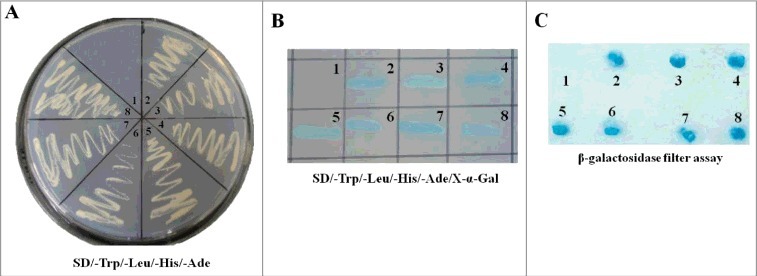
Screening the cellular proteins interacting with NDV M protein through yeast two-hybrid assay. (A) The bait plasmid pGBKT7-M transformed AH109 was mated with yeast Y187 containing pGADT7-Rec with the cDNA library of DF-1 cells. The suspected positive colonies were screened on the auxotrophic medium SD/-Trp/-Leu/-His/-Ade. N0.1 was the negative control and no.2 was the positive control. (B) The yeast colonies obtained from (A) were cultured on the SD/-Trp/-Leu/-His/-Ade/X-α-gal medium. The colonies growing on this medium and turning blue are the indication of interaction of the two expressed proteins. (C) The yeast colonies obtained from (A) were transferred into nitrocellulose filters and assayed for β-galactosidase activity to verify the interaction between NDV M protein and the cellular proteins.

To define cellular proteins that interact with NDV M protein, a yeast two-hybrid screening strategy was employed. The NDV M protein was used as the bait for screening the cDNA library generated from DF-1 cells. The results showed that six colonies (No. 3 to 8) could grow on the auxotrophic medium SD/-Ade/-His/-Trp/-Leu and turn blue in the presence of X-α-gal, which had the same presentation as the positive control transformed with pGBKT7-53 and pGADT7-T (No. 2) (Fig. 1A and B). However, the negative control could neither not grow nor turn blue on the medium SD/-Ade/-His/-Trp/-Leu/X-α-gal (No. 1). To further confirm the true interaction with M protein in yeast, β-galactosidase colony-lift filter assay was performed. We found that yeast colonies co-transformed with the pGADT7-derivative plasmids and pGBKT7-M plasmid all turned blue within 30 min, which was similar to the positive control (Fig. 1C). The nucleotide sequences of six colonies from the cDNA library were then sequenced and analyzed. The results showed that the sequences of six clones had the right open reading frame (ORF) in frame with the AD coding region, and five cellular proteins containing the interaction region were identified by bioinformatics analysis (Table 1). Fortunately, of all these proteins, importin β1 is demonstrated to be a karyopherin that transports multiple proteins owing NLS into the nucleus [23]. Therefore, we conclude that NDV M protein may bind importin β1 to enter the nucleus.

**Table 1.** Comparison between positive clones and similar sequences in GenBank.

Homologous genes (mRNA)	Number of similar clone	GenBank Accession No.	Homologous region	Homology^*^ (%)
Eukaryotic translation elongation factor 2	1 (clone 3)	NM_205368.1	1783-2307 (CDS: 31–2607)	100
Serine and arginine rich splicing factor 3	1 (clone 4)	NM_001195554.1	136-510 (CDS: 115–609)	99
Matrin 3	2 (clone 5 and 8)	NM_204147.1	2017-2460 (CDS: 328–3036)	100
Importin β1	1 (clone 6)	XM_015299473.1	1007-1300 (CDS: 181–2811)	99
Bromodomain containing 2	1 (clone 7)	NM_001030674.1	1717-2016 (CDS: 1–2190)	100

* Homology comparison with Gallus sequences.

### NDV M protein interacts with importin β1 in vivo and in vitro

**Figure 2.**
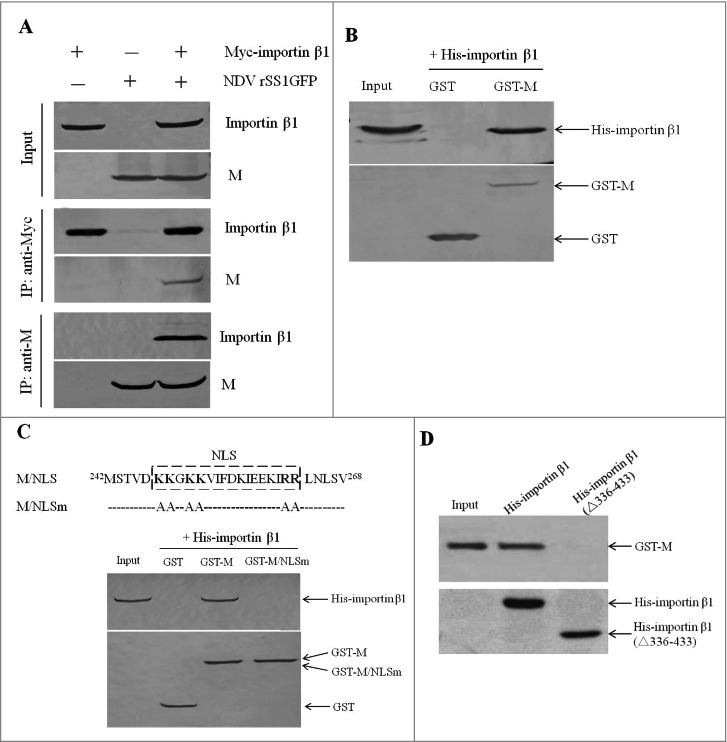
NDV M protein interacts with importin β1 *in vivo* and *in vitro*. (A) Reciprocal co-immunoprecipitation assay of Myc-importin β1 and NDV M protein in DF-1 cells. DF-1 cells transfected with plasmid expressing Myc-importin β1 were infected with NDV at an MOI of 0.1. Cells were lysed at 24 h post-infection, and co-immunoprecipitation assay was performed using either anti-Myc (middle panel) or anti-M (lower panel) antibodies. Immunoprecipitated proteins were detected by Western blotting using anti-M or anti-Myc antibodies. (B) The interaction between M and importin β1 was verified by GST pull-down assay. GST or GST-M or His-importin β1 protein was expressed in *E. coli* BL21 (DE3) and purified on Glutathione-Sepharose beads or His*Bind resins, respectively. The purified GST or GST-M protein (3 μg) was immobilized on Gluthatione-Sepharose beads and then incubated with the purified His-importin β1 (3 μg) for 2 h at 4°C. The beads were washed with transport buffer and the bound proteins were eluted from the beads and detected by Western blotting. (C and D) GST-M/NLSm or His-importin β1(△336-433) was expressed in *E. coli* BL21 (DE3) and then purified as described above. GST pull-down and His pull-down assays were performed to identify the interaction domains between M and importin β1.

To verify the interaction between NDV M protein and importin β1, we first performed co-immunoprecipitation assay with DF-1 cells transiently transfected with plasmid encoding Myc-importin β1 and infected with NDV. Expression of the fusion protein Myc-importin β1 and viral M protein was confirmed with anti-Myc and anti-M antibodies, respectively (Fig. 2A, upper panel). In addition, the M protein and importin β1 in cell supernatants could be immunoprecipitated with each other when using anti-Myc or anti-M antibody (Fig. 2A, middle and lower panels). Next, *in vitro* binding assay of the fusion protein GST-M to the purified His-importin β1 protein showed that His-importin β1 was pulled-down by GST-M protein but not by GST (Fig .2B). These results suggest that that NDV M protein physically interacts with importin β1 protein *in vivo* and *in vitro*.

Previous studies have shown that importin β1 mediates the nuclear import of cargo proteins by binding their NLS [23–25]. Here, the binding studies revealed that the NLS region in the M protein was important for importin β1 binding, since GST-M protein with the mutated NLS lost its binding activity to importin β1 (Fig. 2C). In addition, His-importin β1 deleting residues 336 to 433 (△336-433) could not be pulled down by GST-M (Fig. 2D), indicating that the amino acids 336–433 of importin β1 was essential for interaction with M. It is reported that human importin β1 contains importin-β N-terminal (IBN_N) domain at the N-terminus and several “HEAT repeat” motifs that mostly occupy the C-terminal portion [37]. After comparison with the amino acids of human importin β1, the residues 336 to 433 in chicken belonged to the 8–10 HEAT repeats, which were also the RanGTP binding region. Thus, we demonstrate that the NLS region of NDV M protein and the 8–10 HEAT repeats of importin β1 are important for interaction with each other.

### NLS mutation in the M protein attenuates the replication and pathogenicity of NDV

**Figure 3.**
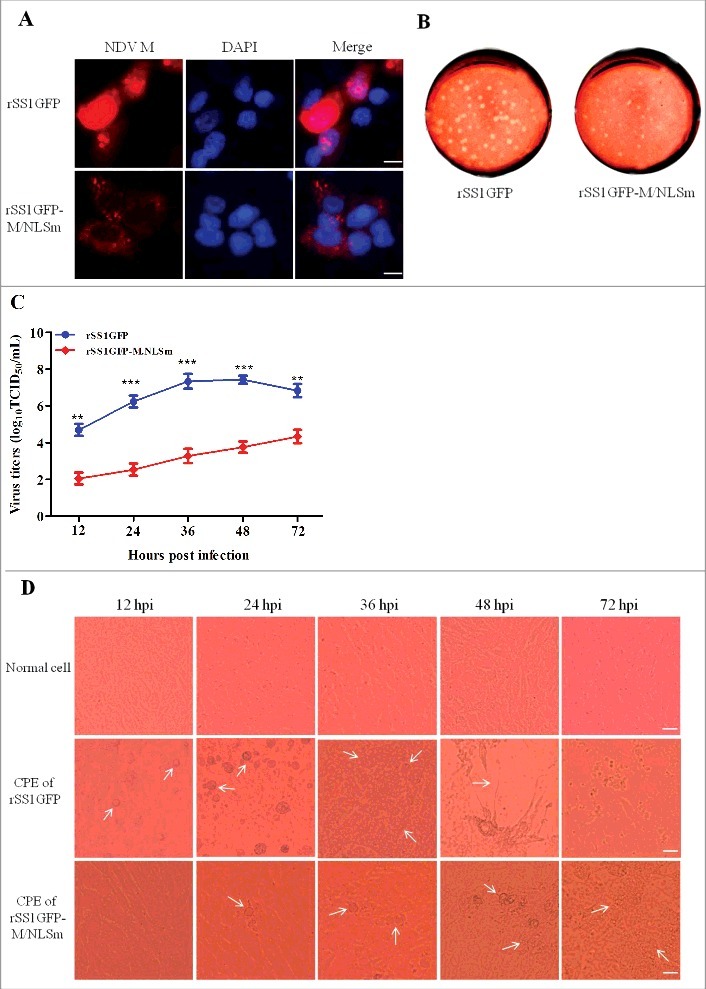
M/NLS mutation reduces the replication ability of NDV in cells. (A) The immunofluorescence assay was used to examine the subcellular localization of NDV M protein in rSS1GFP and rSS1GFP-M/NLSm infected DF-1 cells at 12 hpi. DAPI was used to stain nuclei. Original magnification was 1 × 200. (B) The shape and size of plaques formed by rSS1GFP and rSS1GFP-M/NLSm in DF-1 cells. DF-1 cells in six-well plates were infected with serial tenfold dilutions of the virus for 1 h. After adsorption, the inoculum was removed and replaced with the medium containing 2% FBS and 1% agar. The overlay medium supplemented with 0.1% neutral red was added after 36 h incubation. Plaques were observed after a further 48 h of incubation. (C) Virus titers were determined in DF-1 cells at the indicated time points. Each data point on the curve indicates the mean ± SD of three independent experiments. P values < 0.001 are represent with*** and p values <0.01 are represent with **. (D) The CPE was observed in virus-infected DF-1 cells at the indicated time points. The images were obtained using the phase-contrast microscope. Original magnification was 1 × 200.

Now that the NLS of M protein was essential for its interaction with importin β1, we investigated the effect of M/NLS mutation on the virulence, replication ability and pathogenicity of NDV. The results of virus rescue showed that hemagglutination (HA)-positive allantoic fluid was directly detected in the parental virus rSS1GFP, but three extra egg passages were required for the M/NLS mutant virus rSS1GFP-M/NLSm to be detected by HA test, indicating that the growth of the mutant virus in chicken eggs was slowed down. To determine the stability of M gene mutant virus, the rescued virus rSS1GFP-M/NLSm was plaque purified and passaged five times in 10-day-old specific pathogen free (SPF) chicken eggs. Sequence analysis of the whole-genome of the mutant virus after five passages showed that the introduced M/NLS mutation was unaltered (see Figure S1 in the supplemental material), and no additional mutations were observed in the M gene and other viral genes (data not shown). The immunofluorescence results showed that NLS mutation absolutely disrupted the nuclear localization of M protein in virus-infected cells (Fig. 3A). Meanwhile, the biological characteristics detection revealed that M/NLS mutation significantly extended the MDT of rSS1GFP-M/NLSm (>120 h) in embryonated chicken eggs compared to rSS1GFP (54 ± 2 h), and the ICPI value of rSS1GFP-M/NLSm (1.67 ± 0.01) was lower than that of rSS1GFP (1.88 ± 0.02) (Table 2), indicating that M/NLS mutation could result in a pathotype change of NDV. In addition, the plaques produced by the viruses indicated that cells infected with rSS1GFP developed large and more plaques with a mean size of 2.85 ± 0.35 mm, while cells infected with rSS1GFP-M/NLSm developed much less and smaller plaques with a mean size of 0.87 ± 0.30 mm (Fig. 3B). On the other hand, multicycle growth kinetics experiments revealed that the virus titers of rSS1GFP-M/NLSm were remarkably reduced in comparison to that of rSS1GFP from 12 to 72 hpi (*P*<0.01) (Fig. 3C). Meanwhile, the cytopathic effect (CPE) in rSS1GFP infected cells started at 12 hpi and cell monolayer was absolutely destroyed at 72 hpi, but the CPE in rSS1GFP-M/NLSm infected cells started at 24 hpi and cell monolayer was still existent at 72 hpi (Fig. 3D), which showed much slighter and slower CPE than that of rSS1GFP-infected cells at the same time points.

**Table 2.** Biological characteristics of the parental and mutant viruses^*^.

Virus	Pathogenicity		Virus titer		
	MDT (h)	ICPI	EID_50_/ml	TCID_50_/ml	HA
rSS1GFP	54 ± 2	1.88 ± 0.02	108].67]	108].79]	8 log_2_
rSS1GFP-M/NLSm	>120	1.67 ± 0.01	105].34]	106].17]	4 log_2_

* MDT, mean death time; ICPI, intracerebral pathogenicity index; EID_50_, 50% egg infectious dose; TCID_50_, 50% tissue culture infective dose.

**Figure 4.**
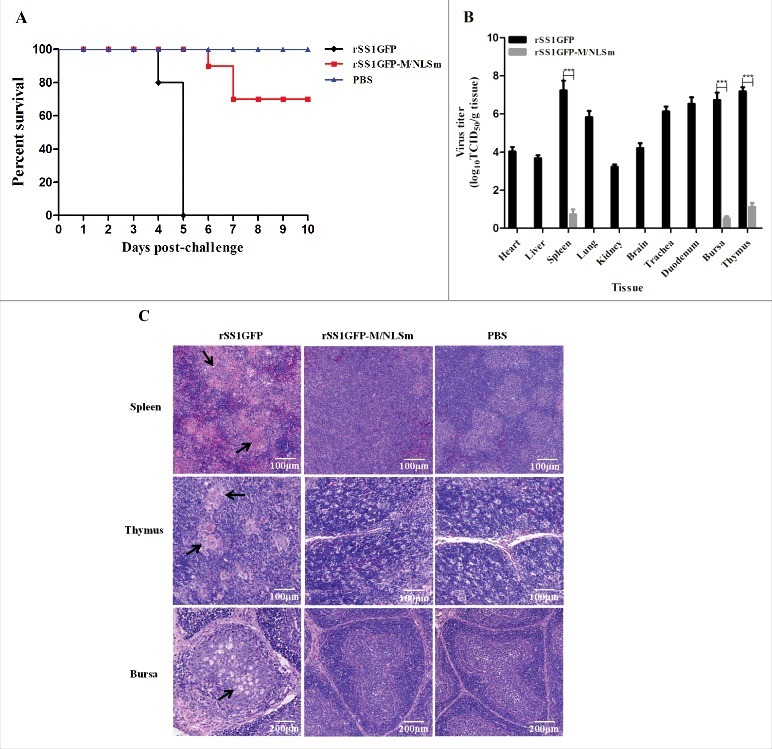
M/NLS mutation attenuates the replication and pathogenicity of NDV in chickens. (A) Survival curves of 4-week-old SPF chickens (n = 10 for each group). Birds were inoculated with rSS1GFP or rSS1GFP-M/NLSm at a dose of 10^5.0^ EID_50_/100 μL per bird, or with 100 μL PBS as the negative control. The birds were monitored for clinical signs daily for 10 dpi. (B) Viral load in the collected tissues of 4-week-old chickens (n = 3) infected with the two viruses at 5 dpi. Viral titers were determined in DF-1 cells and presented as log_10_TCID_50_ g^−1^ tissue. Asterisks indicate a statistically significant difference between the titers of the two viruses. P values < 0.001 are represent with***. (C) Histopathology of lymphoid tissue samples collected from rSS1GFP or rSS1GFP-M/NLSm or PBS-inoculated 4-week-old chickens. Birds were sacrificed at 5 dpi, and the tissues were fixed with 10% neutral formalin, sectioned, and stained with hematoxylin-eosin.

The *in vivo* pathogenesis assessment of M/NLS mutant virus rSS1GFP-M/NLSm in 4-week-old SPF chickens was then evaluated. The resulting survival curves were shown in Fig. 4A. Birds inoculated with the parental virus rSS1GFP exhibited slight depression at 3 days post-infection (dpi), severe depression (3/10), wing drop (2/10), hemiparesis/paralysis (3/10), and death (2/10) at 4 dpi, and 100% mortality by 5 dpi. At necropsy, all euthanized chickens presented severe gross lesions in multiple organs, such as conjunctivitis, hemorrhage of the throat, trachea, thymus, duodenum mucosa, and proventriculus, multifocal necrosis of the spleen, and marked atrophy of thymus and bursa of Fabricius at 4–5 dpi. In comparison, birds inoculated with the mutant virus rSS1GFP-M/NLSm presented delayed and slight depression (4/10) at 5 dpi, severe depression (2/10) and one death (1/10) at 6 dpi, and two death (2/10) at 7 dpi, but there were no death in the subsequent days. Virus titration assays showed that the mutant virus rSS1GFP-M/NLSm had little replication ability in spleen, thymus and bursa of Fabricius at 5 dpi; by contrast, the parental virus rSS1GFP replicated in multiple tissues and had relatively higher virus titers in the lymphoid tissues (spleen, thymus and bursa of Fabricius) (Fig. 4B). In addition, the results of histopathology observation of the lymphoid tissues showed that birds inoculated with rSS1GFP displayed multifocal confluent coagulative necrosis, severe lymphocyte depletion, and infiltration of macrophages, whereas no apparent histopathological changes were observed in the lymphoid organs of the rSS1GFP-M/NLSm group and the control group (Fig. 4C). Taken together, these results clearly demonstrate that M/NLS mutation can not only reduce viral replication ability in chicken fibroblasts but also attenuate viral replication and pathogenicity in SPF chickens.

### Nuclear import of NDV M protein requires importin β1 and RanGTP

**Figure 5.**
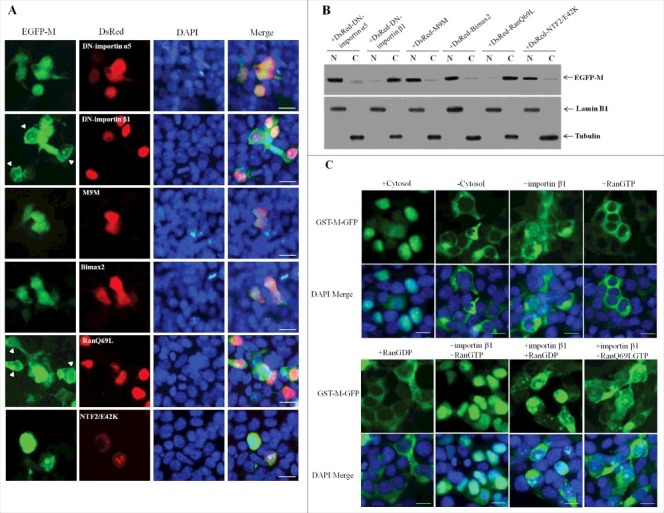
Nuclear import of NDV M protein requires importin β1 and RanGTP. (A) DF-1 cells were transiently co-transfected with the plasmid pEGFP-M and plasmids encoding DsRed-DN-importin α5, DsRed-DN-importin β1, DsRed-M9M, DsRed-Bimax2, DsRed-RanQ69L or DsRed-NTF2/E42K, respectively. The subcellular localization of the fusion proteins was observed under fluorescence microscope at 24 h post-transfection. DAPI was used to stain nuclei. Original magnification was 1 × 200. (B) The intracellular distribution of the fusion protein EGFP-M obtained from (A) was detected by Western blotting. Lamin B1 for the nucleus and tubulin for the cytoplasm were used as cellular markers. N represents the nucleus and C represents the cytoplasm. (C) Digitonin-permeabilized HeLa cells were incubated with GST-M-GFP in the presence of cytosol, importin β1, RanGTP, RanGDP, importin β1 plus RanGTP, importin β1 plus RanGDP, or importin β1 plus RanQ69LGTP. DAPI was used to stain nuclei. The GST-M-GFP protein was observed under fluorescence microscope. Original magnification was 1 × 200.

Numerous studies have shown that importin β1 together with RanGDP and/or RanGTP participate in the nuclear import of many cargo proteins [38–42]. To identify the cellular karyopherins responsible for M nuclear targeting and further verify the nuclear import pathway of NDV M protein, two dominant-negative (DN) mutants of importin α5 (DN-importin α5) [43] and importin β1 (DN-importin β1) [44], which lack the ability to bind importin β and Ran, respectively, and nuclear import inhibitors M9M [45] or Bimax2^46^ that are specific for the transportin-1 pathway or the importin α1, α3, α6 and α7 pathways, respectively, RanGTP mutant (Ran/Q69L) [47], which is deficient in GTP hydrolysis, and NTF2 mutant (NTF2/E42K) [48,49], which fails to transport RanGDP into nucleus, were first introduced to determine whether they are required for the nuclear import of NDV M protein. As shown in Fig. 5A, DF-1 cells co-transfected with plasmid pEGFP-M and plasmids encoding DsRed-DN-importin α5 or DsRed-M9M or DsRed-Bimax2 or DsRed-NTF2/E42K did not impair the nuclear localization of EGFP-M, while co-expression of either DsRed-DN-importin β1 or DsRed-Ran/Q69L inhibited the nuclear accumulation of EGFP-M. In addition, detection of the intracellular localization of EGFP-M by Western blotting also verified that the EGFP-M had the same distribution as the fluorescence microscopy (Fig. 5B).

*In vitro* nuclear import assay was then performed to further confirm that importin β1 and RanGTP are sufficient for the nuclear import pathway of NDV M protein. The results showed that the fusion protein GST-M-GFP was efficiently imported into the nucleus of digitonin-permeabilized HeLa cells in the presence of exogenous cytosol (Fig. 5C). However, GST-M-GFP showed no nuclear translocation with RanGTP or RanGDP alone, which is similar to the results observed in the absence of cytosol, whereas combination of purified importin β1 plus RanGTP exhibited a much stronger level of nuclear accumulation than that seen when the cytosol was added (Fig. 5C). Importantly, importin β1 plus RanGDP or importin β1 alone or replacement of RanGTP with RanQ69LGTP (importin β1 plus RanQ69LGTP) led to a small amount of nuclear accumulation of GST-M-GFP (Fig. 5C). Therefore, these results confirm that importin β1 together with RanGTP but not RanGDP are the components required to mediate the active nuclear import of NDV M protein, and this process depends on GTP hydrolysis by Ran.

### Nuclear import of NDV M protein does not require importin α

**Figure 6.**
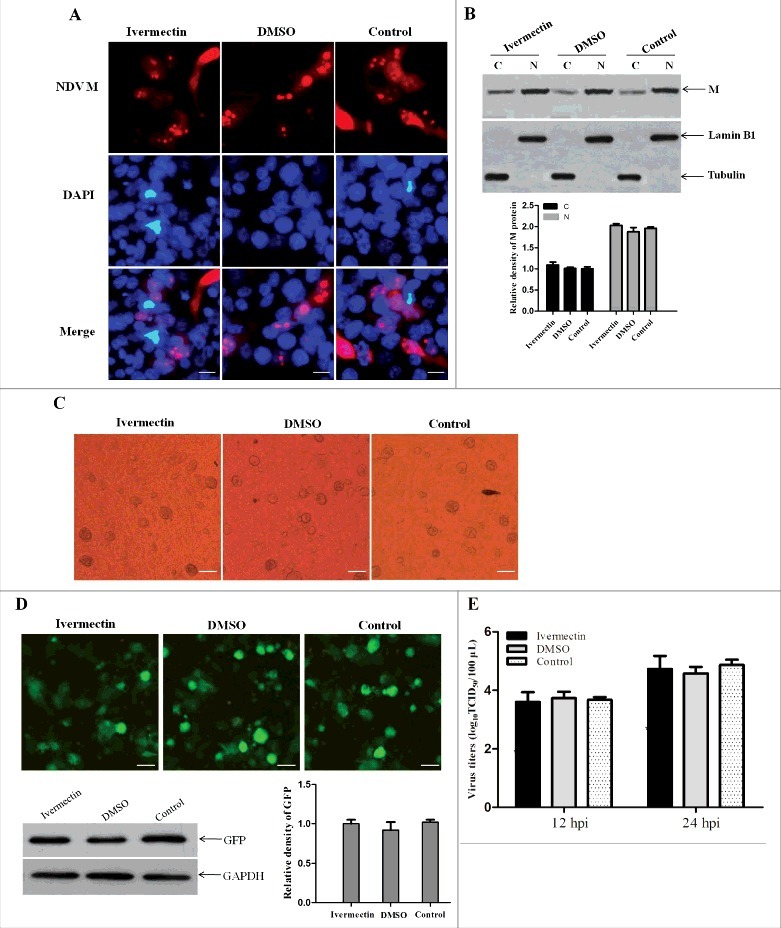
The nuclear import of M protein and the replication of NDV are not inhibited by ivermectin. (A) The subcellular localization of NDV M protein in virus-infected DF-1 cells treated with ivermectin or DMSO at 12 hpi. DAPI was used to stain nuclei. Original magnification was 1 × 200. (B) The intracellular distribution of NDV M protein obtained from (A) was detected by Western blotting. Lamin B1 for the nucleus and tubulin for the cytoplasm were used as cellular markers. N represents the nucleus and C represents the cytoplasm. (C) The CPE in virus-infected DF-1 cells treated with ivermectin or DMSO at 12 hpi was observed under phase-contrast microscope. Original magnification was 1 × 200. (D) The expression of GFP in virus-infected DF-1 cells was observed under fluorescence microscope and detected by Western blotting. (E) DF-1 cells were treated with the drug ivermectin or DMSO and then infected with rSS1GFP at an MOI of 0.1. The cell culture supernatants were collected at 12 and 24 hpi, and the virus titers were determined as TCID_50_ in DF-1 cells.

Ivermectin is reported to be specific for importin α/β1-mediated nuclear import of cargoes, and has no effect on any of the other nuclear import pathways including that mediated by importin β1 alone [50]. Therefore, ivermectin was used to further verify whether NDV M protein can be imported into the nucleus without the participation of importin α. We found that there was no obvious difference in the subcellular localization of NDV M protein in rSS1GFP-infected DF-1 cells treated with ivermectin or DMSO when compared to the untreated cells at 12 hpi (Fig. 6A and B). In addition, the ivermectin- or DMSO-treated DF-1 cells also had the same characteristics in the generation of CPE and the expression of GFP when infected with rSS1GFP (Fig. 6C and D). Moreover, the replication capacity of rSS1GFP was not affected in either ivermectin or DMSO treated cells at 12 hpi and 24 hpi (Fig. 6E). These results demonstrate that nuclear import of M protein and the replication of NDV are not inhibited by ivermectin, and therefore do not require importin α.

### Importin α5 interacts with NDV M protein by binding importin β1

**Figure 7.**
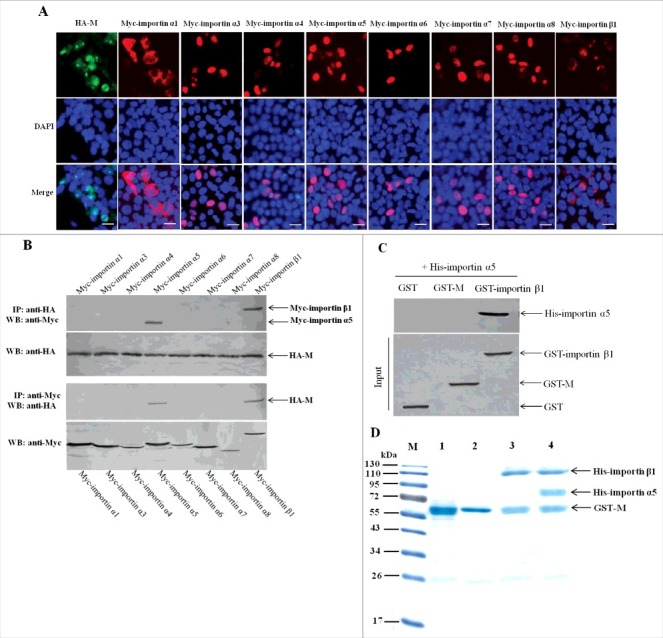
Importin α5 binds NDV M protein by interacting with importin β1. (A) The subcellular localization of the fusion proteins in DF-1 cells. The indicated plasmids were transfected into DF-1 cells and then used for immunofluorescence aasay at 24 h post-transfection. DAPI was used to stain nuclei. Fluorescent images were obtained under a Nikon fluorescence microscope. Original magnification was 1 × 200. (B) Characterization of the interaction between NDV M protein and the cellular transport proteins by reciprocal co-immunoprecipitation assay. DF-1 cells transfected with the plasmids were lysed at 24 h post-transfection, and co-immunoprecipitation assay was performed using either anti-HA (upper panel) or anti-Myc (lower panel) antibodies. Immunoprecipitated proteins were detected by Western blotting using anti-Myc or anti-HA antibodies. (C) Identification of the interaction between importin α5 and M or importin β1 by pull-down assay. The purified GST-M or GST-importin β1 protein was immobilized on Gluthatione-Sepharose beads and then incubated with the purified His-importin α5. The bound proteins were eluted from the beads and examined by Western blotting. (D) Protein binding assay was used to identify the interaction among M, importin α5 and importin β1. Lane 1 was GST-M alone, lane 2 was GST-M plus His-importin α5, lane 3 was GST-M plus His-importin β1, lane 4 was GST-M plus His-importin α5 together with His-importin β1.

Because some of the importin α members can act as negative regulators for importin β1-mediated nuclear import of cargo proteins, so we wanted to search for the potential importin α that may indirectly bind NDV M protein and study its role in the nuclear import process of NDV M protein. Up to now, there are seven importin α members including importin α1, α3, α4, α5, α6, α7 and α8 have been identified in humans and many animals [51–53]. The immunofluorescence assay was first performed to examine the subcellular localization of these fusion proteins expressed by the recombinant eukaryotic expression vectors. As shown in Fig. 7A, besides the cytoplasmic and nuclear envelope localization of Myc-importin α1 and Myc-importin β1, the other fusion proteins including Myc-importin α3 to Myc-importin α8 and HA-M exhibited the similar localization in the nucleus. Next, the co-immunoprecipitation and pull-down assays were used to identify the potential importin α proteins that interact with NDV M protein. The results showed that both Myc-importin α5 and Myc-importin β1 could be immunoprecipitated by HA-M (Fig. 7B). However, the pull-down assay indicated that GST-importin β1 but not GST-M could be pulled-down by His-importin α5 (Fig. 7C). Because endogenous importin α5 can interact with importin β1 in the process of transporting cargo proteins into the nucleus [21, 22], we investigated if NDV M protein can directly bind importin α5. The results of subsequent protein-binding assays showed that when GST-M immobilized on Glutathione-Sepharose beads was incubated with His-importin α5 or His-importin β1, His-importin β1 but not His-importin α5 was pulled-down by GST-M (Fig. 7D, lanes 2 and 3). However, when immobilized GST-M was incubated simultaneously with His-importin α5 and His-importin β1, both His-importin α5 and His-importin β1 were pulled-down by GST-M and detected by SDS-PAGE (Fig. 7D, lane 4). Therefore, these results demonstrate that importin α5 interacts with NDV M protein by binding importin β1.

### Importin α5 reduces importin β1-mediated nuclear import of NDV M protein

**Figure 8.**
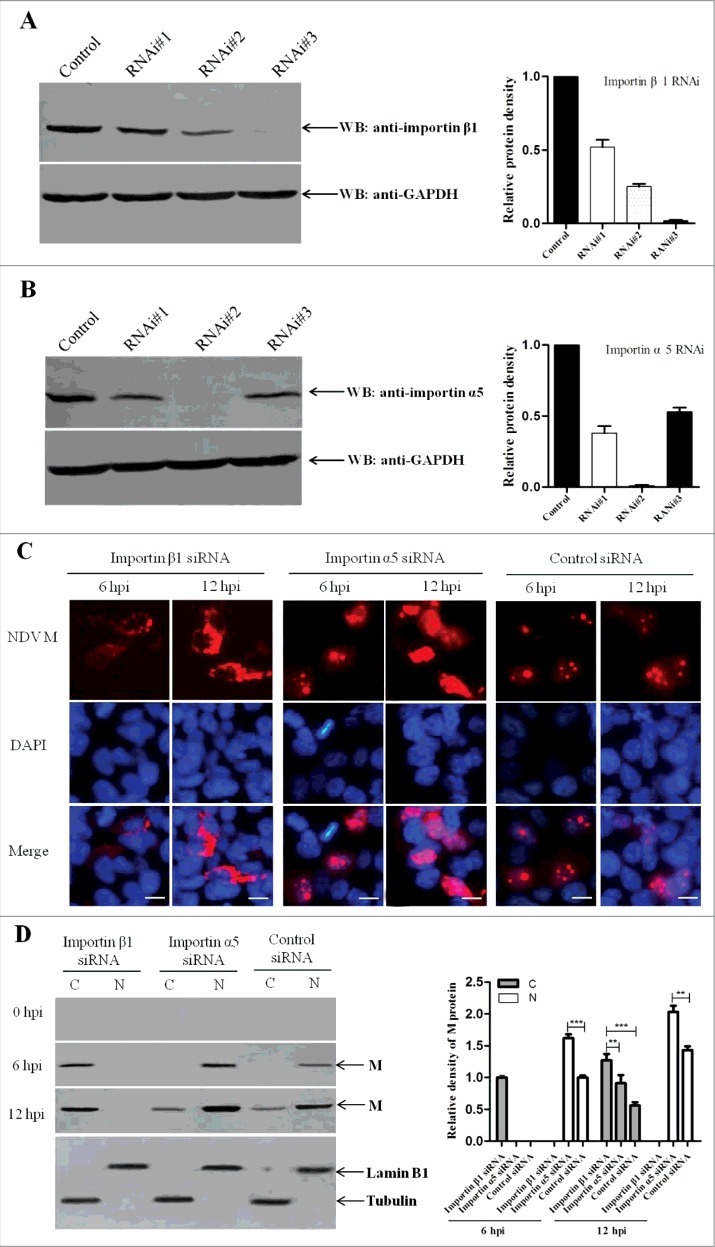
Importin α5 reduces importin β1-mediated nuclear import of NDV M protein. (A and B) Effects of importin β1 or importin α5 RNAi on the expression of endogenous importin β1 or importin α5, respectively. DF-1 cells were transfected with importin β1 or importin α5 siRNA (#1-3) or control siRNA. After 48 h transfection, cell lysates were prepared and examined by Western blot with anti-importin β1 or anti-importin α5 antibody. Endogenous GAPDH expression was used as internal control. (C) DF-1 cells were transfected with importin β1 siRNA or importin α5 siRNA or control siRNA. Forty-eight hours after transfection, cells were infected with rSS1GFP at an MOI of 1. The subcellular localization of NDV M protein in virus-infected DF-1 cells was detected at 6 hpi and 12 hpi, respectively. DAPI was used to stain nuclei. Original magnification was 1 × 200. (D) Immunoblot analysis of the intracellular distribution of M in importin β1 siRNA- or importin α5 siRNA- or control siRNA-treated DF-1 cells. N represents the nucleus and C represents the cytoplasm.

According to the above results and for the reason that importin α5 can act as negative regulator in importin β1-mediated nuclear import pathway, we hypothesized that importin α5 might play the inhibition function in the subcellular localization of NDV M protein. To test this hypothesis, the localization of M protein in importin β1- or importin α5-depleted DF-1 cells was investigated. Three pairs of synthesized importin β1 or importin α5 siRNAs (see Table S1 in the supplemental material) were transfected into DF-1 cells respectively, and importin β1 RNAi#3 or importin α5 RNAi#2 could effectively lower the expression level of importin β1 or importin α5 without causing discernable changes in cell morphology (Fig. 8A and B). In addition, the viability of those cells receiving siRNA using trypan blue exclusion and MTT assays were examined. Results showed that there was no difference between importin β1 or importin α5 RNAi and RNAi control in terms of viability of transfected cells (data not shown). We then infected DF-1 cells receiving the indicated siRNA or control siRNA with NDV strain rSS1GFP. As a result, at 6 or 12 hpi, importin β1 depletion caused M protein mainly in the cytoplasm, further confirming that importin β1 mediated the nuclear import of M protein. However, importin α5 depletion markedly increased the nuclear accumulation of M protein when compared to that of control siRNA group (Fig. 8C). Similar results were obtained by examining the intracellular distribution of M protein in importin β1 siRNA- or importin α5 siRNA- or control siRNA-treated cells by immunoblot analysis (Fig. 8D). Together with the above results, these data reveal that the importin β1-mediated nuclear import of NDV M protein is reduced in the presence of importin α5.

### Importin α5 decreases importin β1-participted NDV replication and pathogenicity

**Figure 9.**
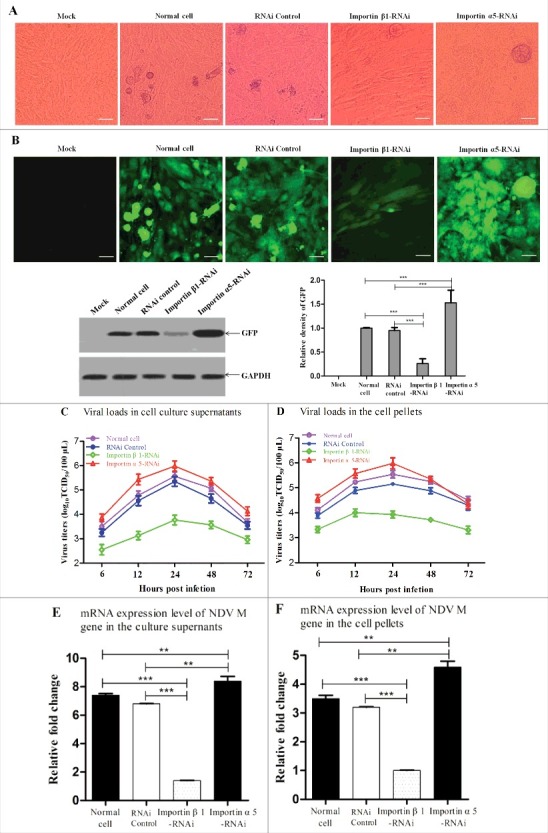
Importin α5 decreases importin β1-participated NDV replication and pathogenicity. (A) Normal cells, RNAi control cells, importin β1-RNAi cells and importin α5-RNAi cells were infected with rSS1GFP at an MOI of 1. Twenty-four hours after infection, the CPE was observed under phase-contrast microscope. (B) The expression of GFP obtained from (A) was observed under fluorescence microscope and detected by Western blotting. (C and D) Normal cells, RNAi control cells, importin β1-RNAi cells and importin α5-RNAi cells were infected with rSS1GFP at an MOI of 1. At different time points (6, 12, 24, 48 and 72 hpi), the viral loads in the cell culture supernatants (C) and cell pellets (D) were determined by TCID_50_ in DF-1 cells. The graphs showed the average of viral titers in DF-1 cells from three independent experiments. (E and F) qRT-PCR was used to examine the mRNA expression levels of NDV M gene in the cell culture supernatants (E) and cell pellets (F) obtained from (C) and (D) at 24 h post-infection, respectively. The graphs showed the average of mRNA levels of NDV M gene in DF-1 cells from three independent experiments. P values < 0.001 are represent with*** and p values <0.01 are represent with **.

Since importin α5 causes the reduction of nuclear localization of M protein, this might affect the replication and pathogenicity of NDV. To this end, siRNA-mediated knockdown of importin β1 or importin α5 in DF-1 cells infected with NDV strain rSS1GFP was investigated. As shown in Fig. 9A and B, the normal cells and control siRNA-treated cells showed the similar CPE and GFP expression level at 24 hpi, while siRNA-mediated knockdown of importin β1 markedly reduced NDV-induced CPE and GFP expression level, but knockdown of importin α5 greatly enhanced the CPE and GFP expression level when compared to the normal cell group and RNAi control group. In addition, significant reduction of viral loads in the cell culture supernatants and cell pellets of importin β1-RNAi cells was detected, but an increasing of viral loads in that of importin α5-RNAi cells were examined in comparison to that of normal cell and RNAi control (Fig. 9C and D). To further determine the effect of importin β1 or importin α5 knockdown on NDV replication, the mRNA expression levels of NDV M gene in NDV-infected importin β1-RNAi or importin α5-RNAi cells were examined at 24 hpi. We found that the mRNA expression level of NDV M gene in importin β1-RNAi or importin α5-RNAi cells significantly decreased or increased , respectively, in comparison to that of normal cells and RNAi control either in the culture supernatants or in the cell pellets (*P* < 0.001) (Fig. 9E and F). Together, above results demonstrate that importin α5 acts as negative regulator in importin β1-participated NDV replication and pathogenicity in cells.

## Discussion

Paramyxoviruses are a diverse group of enveloped viruses with non-segmented negative-sense single-stranded RNA genomes that include a number of important human and animal pathogens [5]. To date, the pathogenic mechanism of the paramyxoviruses still attracts the global researchers’ attentions. Two envelope glycoproteins, the attachment protein (termed HN for hemagglutinin-neuraminidase, H for hemagglutinin, or G for glycoprotein, depending on the virus) and the fusion (F) protein, have been always reported to be the major virulence and pathogenic factors for paramyxoviruses [54–56]. However, in recent years, increasing number of researches has focused on the M protein in the pathogenesis of paramyxoviruses due to its multifunction in inhibiting the host RNA and protein synthesis and facilitating the assembly and budding of progeny virions [57–59]. Among the members of paramyxoviruses, the M protein of HRSV, SeV, NiV and NDV is demonstrated to shuttle between the nucleus and cytoplasm through intrinsic NLS and NES [5]. Up to now, only the nucleocytoplasmic shuttling mechanism and the detailed function of HRSV M protein are elucidated [35,36]. We previously have demonstrated that the nuclear export of NDV M protein is mediated by three NESs via the CRM1-independent pathway [60], but the nuclear import mechanism of NDV M protein and the definite functions of M protein in the nucleus are still not known.

Numerous studies have revealed that NLS-mediated nuclear localization of viral proteins is crucial for the replication and pathogenicity of most viruses [28–31]. We previously found that a basic amino acid mutation, R42A, in the NDV M protein not only abrogates its nuclear localization but also attenuates NDV replication and pathogenicity [61]. However, R42A mutation in the M protein does not really reflect the effect of nuclear localization disruption of M protein on the replication and pathogenicity of NDV. Here, we successfully rescued the M/NLS mutant virus rSS1GFP-M/NLSm after three extra chicken egg passages and found that the disruption of M's nuclear localization caused by M/NLS mutation not only resulted in a pathotype change of NDV but also reduced viral replication in cells and attenuated the replication and pathogenicity of NDV in SPF chickens, thus demonstrating for the first time the importance of M's nuclear localization for NDV replication and pathogenicity. In addition, the rescued viruses rSS1GFP and rSS1GFP-M/NLSm could be used to further investigate the more detailed functions of M's nuclear localization in the pathogenesis of NDV utilizing transcriptomics and proteomics. However, it was strange that the MDT and ICPI values of rSS1GFP-M/NLSm did not correlate, which showed that the MDT value suggested the virus was lentogenic, but the ICPI value suggested the virus was velogenic. In our previous studies, we also found that there was no relevance in the MDT and ICPI values of the mutant virus carrying M/F23A (MDT was 102 h, ICPI was 1.64) or M/P24A (MDT was 84 h, ICPI was 1.70) or M/R42A (MDT was 115 h, ICPI was 1.64) when compared to the parental virus rZJ1GFP (MDT was 52 h, ICPI was 1.91) [61,62]. Another research group has studied the effect of some conserved amino acids mutation in the HN protein on the virulence of NDV. They similarly find that in comparison to the parental virus (MDT is 62 h, ICPI is 1.51), the MDT of Y526Q mutant NDV increases to 98 h, which belongs to the lentogen strain, whereas the ICPI value (1.33) of the virus indicates that it is still mesogenic strain [63]. We speculated that if the amino acid sequences at the F protein cleavage site that determine the virulence characteristics of NDV are not changed, mutating certain key amino acids in viral proteins will probably not make the MDT and ICPI values correlate, but the ICPI values of the mutant viruses still keep the original virulence characteristics.

In recent years, cellular nuclear transport receptor proteins-mediated nuclear import of viral proteins has been always the spotlight. In the present study, importin β1 was identified to mediate the nuclear import of NDV M protein by binding its NLS region via the RanGTP-dependent pathway. Numerous studies have shown that Arg/Lys-rich NLSs within cargo proteins are the binding sites for the recognition and binding of importin α or importin β [20–24]. Generally, classical NLSs including monopartite and bipartite NLSs are transported into the nucleus by importin α/β heterodimer, whereas non-classical NLSs can be more complex in sequence, length and amino acid composition that are imported by importin β [22,24]. However, large numbers of studies have found that classical NLSs can also be recognized and bound by importin β1, such as the NLS of HTLV-1 Rex (RRRPRRSQRKR) [64], HIV-1 Tat (RKKRRQRRR) and Rev (RQARRNRRRR) [65], Smad3 (KKLKK) [66], TopBP1 (RKRK) [67], and BLM (RSKRRK) [68]. It is reported that NDV M protein localizes to the nucleus via a bipartite NLS (KKGKKVIFDKIEEKIRR) [17]. Interestingly, our results similarly demonstrated that the classical NLS of NDV M protein was critical for interaction with importin β1 without preferentially binded to importin α. It has been demonstrated that various types of importin αs are expressed at widely divergent levels in different tissues and show very different affinities for distinct NLSs [69, 70]. Therefore, we concluded that efficient nuclear import of NDV M protein in various kinds of tissues could be achieved by binding importin β1 directly, rather than relying on one or more patterns of importin α as an intermediary.

Previous study has indicated that importin β1 contains two major domains, including importin-β N-terminal (IBN_N) domain at the N-terminus and multiple “HEAT repeat” regions that mostly occupy the C-terminus [37]. Of which the HEAT repeats have the ability to form different conformations in different functional states that facilitate the accommodation of their binding partners by an induced fit type of mechanism [71,72]. Several studies have confirmed that the HEAT repeats of importin β1 can provide abundant binding regions for interaction with distinct cargo proteins. For example, the cellular proteins PTHrP [73], Snail [74], BLM [68], SREBP2 [75] and TopBP1 [67] interact with importin β1 by binding the 2–11, 5–14, 14–16, 7–17 and 18–19 HEAT repeats of importin β1, respectively. Although the HEAT repeats used to bind three cargo proteins (2–11 for PTHrP, 5–14 for Snail, and 7–17 for SREBP2) is overlapped, the binding mechanism for each protein is distinctly different [72]. But anyway, the binding regions of these cargo proteins all overlap with the RanGTP binding region of importin β1 (the 8–10 HEAT repeats), indicating that the formation of cargo protein-importin β1 heterodimer requires a large contact area and the ability for the binary complex to be disassembled by RanGTP binding upon entry to the nucleus. This is in agreement with the previous obtained results [73–75]. Similar to the above findings, we found that the HEAT repeats 8–10 of importin β1 was responsible for interaction with NDV M protein, and the nuclear import of M protein was dependent on RanGTP, further confirming that cargo proteins that interact with the RanGTP binding region of importin β1 require the RanGTP for nuclear targeting.

**Figure 10.**
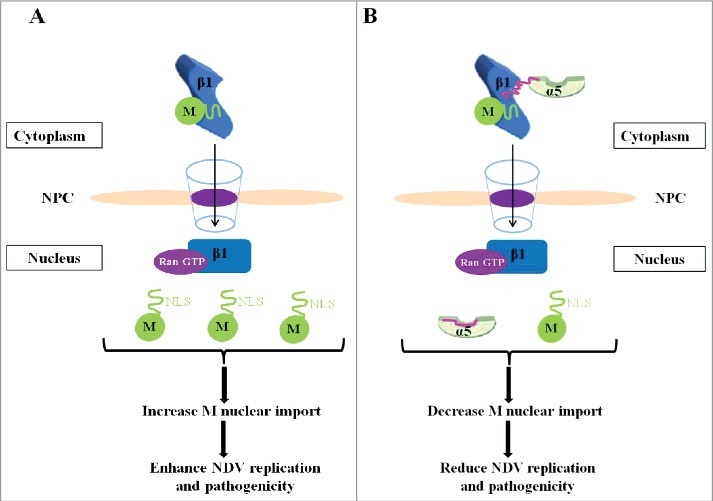
The schematic diagram of the negative regulation role of importin α5 in importin β1-mediated nuclear import of M protein and the replication and pathogenicity of NDV.

Although importin α/β1 heterodimer-mediated nuclear import is thought to be widely used in cells [22], but some studies find that importin α protein can negatively regulate the nuclear import of cargo proteins mediated by importin β1 [26,27]. The action mechanism shows that importin α interacts with the domain of cargo proteins to compete with the binding of importin β1 [26], or forms a ternary complex with the cargo protein-importin β1 complex to reduce the nuclear transport process [27]. In this study, we demonstrated that importin α5 interacted with the importin β1-M binary complex to decrease the nuclear import efficiency of NDV M protein, for the reason that importin α5 directly binded importin β1 but not M protein, and depletion of importin α5 remarkably increased the nuclear accumulation of M protein. In addition, we found that siRNA-mediated knockdown of importin α5 in DF-1 cells infected with NDV caused more serious CPE and more increased viral replication ability in comparison to that of normal cells and RNAi control cells. On the contrary, importin β1 depletion not only disrupted the nuclear localization of M protein but also greatly reduced the CPE induced by NDV infection and viral replication capacity. Moreover, we also found that knockdown of importin β1 decreased the expression levels of NDV M protein and GFP in cells. It has been shown that the nuclear localization of paramyxovirus M protein has two main functions: (i) inhibit host gene transcription and protein synthesis, and (ii) ensure that the replication and transcription of viral genome in the cytoplasm proceed until a certain level of viral protein and RNA expression is reached, at which point M is transported into the cytoplasm to participate in virus assembly [57]. Therefore, we speculated that the disruption of M's nuclear localization could not achieve the above functions and led to the reduction of the replication and transcription of viral genome during the course of virus infection. But more experiments were needed to verify the definite nuclear localization functions of NDV M protein. Together with the above results, our studies clearly demonstrated that importin β1 and RanGTP were responsible for the nuclear import process of NDV M protein, and importin β1 alone could increase the nuclear import efficiency of M protein and enhance NDV replication and pathogenicity (Fig. 10A), whereas such case could be decreased in the presence of importin α5 (Fig. 10B).

In summary, we demonstrated for the first time the nuclear import mechanism of NDV M protein and the negative regulation role of importin α5 in importin β1-mediated nuclear import of M protein and the replication and pathogenicity of NDV. Our results will provide a better understanding of the exact role of M's nuclear localization in NDV life cycle and aid in understanding the poorly understood NDV pathogenesis.

## Materials and methods

### Cells and antibodies

The chicken embryonic fibroblast cell line (DF-1) and HeLa cells were purchased from cell resource center of Shanghai Institutes for Biological Sciences of the Chinese Academy of Sciences. BHK-21 cells (clone BSR-T7/5), generated by Buchholz *et al.* [76], were a kind gift from Professor Zhigao Bu (Harbin Veterinary Research Institute, China). Primary antibodies goat anti-importin α5 polyclonal antibody (sc-6918), rabbit anti-importin β1 polyclonal antibody (sc-11367), mouse anti-GST monoclonal antibody (sc-374171), mouse anti-His monoclonal antibody (sc-8036), mouse anti-GAPDH monoclonal antibody (sc-66163) were purchased from Santa Cruz Biotechnology (USA). Mouse anti-GFP monoclonal antibody (ab1218), mouse anti-HA monoclonal antibody (ab18181), rabbit anti-Myc monoclonal antibody (ab32072), rabbit anti-tubulin polyclonal antibody (ab125267) and rabbit anti-Lamin B1 polyclonal antibody (ab16048) were purchased from Abcam (UK).

### Plasmids construction

All enzymes used for cloning procedures were purchased from Thermo Scientific Company. The ORF of M gene was amplified from the cDNA derived from the virulent genotype Ⅶd NDV strain Sheldrake duck/China/Guizhou/SS1/2014 (SS1) (GenBank no.KP742770) and then subcloned into pGBKT7, pEGFP-C1, pGEX-6p-1, pCMV-HA, pGEX-GFP to generate pGBKT7-M, pEGFP-M, pGEX-6p-M, pCMV-HA-M and pGEX-M-GFP, respectively. The chicken importin β1 or importin α5 ORF was amplified from the cDNA derived from DF-1 cells and then used to construct the plasmids pET-32a-importin β1, pCMV-Myc-importin β1, pGEX-6p-importin β1 and pET-32a-importin α5, respectively. Annealed oligonucleotides encoding the M9M [45] or Bimax2 [46] were inserted into pDsRed-C1 (Clontech) to generate plasmids pDsRed-M9M and pDsRed-Bimax2, respectively. Dominant negative (DN) mutant DN importin α5, DN mutant NTF2 (NTF2/E42K), DN importin β1 or RanQ69L was subcloned into pDsRed-C1 to yield pDsRed-DN-importin α5, pDsRed-DN-NTF2/E42K, pDsRed-DN-importin β1 and pDsRed-RanQ69L, respectively.

To generate the recombinant infectious clones harboring alanine (A) substitution targeting basic amino acid residues in the NLS motif (^247^AAGAAVIFDKIEEKIAA^263^) of M protein, the fragment containing the restriction enzyme sties *Age*Ⅰ and *Bst*Z17Ⅰwas amplified by two pairs of specific primers, which were used to introduce multiple amino acid substitutions in the M protein, to generate two overlapping PCR fragments. The two PCR fragments were joined in a second PCR and the obtained fragment was digested with *Age*Ⅰ and *Bst*Z17Ⅰ to replace the corresponding region in the full-length cDNA clone pNDV/SS1GFP (see Figure S2 in the supplemental material) [77]. The resulting plasmid was named pNDV/SS1GFP-M/NLSm. All the recombinant plasmids were confirmed by PCR, restriction digestion and DNA sequencing. Primers used in this study are available upon request.

### Yeast two-hybrid screening and colony-lift filter assay

The yeast AH109 containing bait plasmid pGBKT7-M was grown on SD/-Trp/X-α-gal, SD/-Trp/-His/X-α-gal and SD/-Trp/-Ade/X-α-gal to exclude the autonomous transcriptional activity. Then the transformed AH109 mated with yeast Y187 containing pGADT7-Rec with the cDNA library of DF-1 cells for 24 h. The screening was performed according to the manufacturer's instructions (Matchmaker™ GAL4 Two-Hybrid System 3) and as previously described [78]. In β-galactosidase colony-filter assay, the prey plasmids in the suspected positive clones were rescued and then co-transformed into AH109 with the plasmid pGBKT7-M. Positive clones grown on SD/-Ade/-His/-Trp/-Leu medium were tested for β-galactosidase activity. The pGBKT7-53 and pGADT7-T or pGBKT7-Lam and pGADT7-T co-transformed group was used as a positive and negative control, respectively. Yeast colonies co-transformed with pGBKT7-M and the pGADT7-derivative plasmids were checked periodically for the appearance of blue colonies. Yeast plasmid was isolated from blue colonies as previously described [79], and the inserted fragment was obtained by PCR amplification and then analyzed by bioinformatics methods.

### Cell culture, transfection and fluorescence microscopy

DF-1 cells were cultured in Dulbecco's modified Eagle's medium (DMEM, GIBCO) containing 10% fetal bovine serum (FBS, GIBCO) supplemented with 100 U/ml penicillin and 100 μg/ml streptomycin at 37°C under an atmosphere with 5% CO_2_. For the transfection experiments, 4 × 10^5^ DF-1 cells were grown to 80% confluence in 35-mm-diameter dishes and then double or single transfected with a total of 3 μg plasmid using the FuGENE HD Transfection Reagent (Roche) according to the manufacturer's recommendations. Twenty-four hours after transfection, cells expressing the fluorescence-fused proteins were rinsed with phosphate-buffered saline (PBS), fixed with 4% paraformaldehyde for 20 min, permeabilized with 0.25% Triton X-100 in PBS for 5 min, and then counterstained with DAPI (Sigma) to detect the nuclei. Fluorescent images were obtained under a Nikon fluorescence microscope (Japan). Analysis and merging of the images were done with Adobe Photoshop 7.0 software.

### Protein interaction assays

For co-immunoprecipitation assay, 4 × 10^5^ DF-1 cells grown in 35-mm-diameter dishes were transfected with plasmid pCMV-Myc-importin β1 for 24 h and then infected with NDV strain rSS1GFP at a multiplicity of infection (MOI) of 0.1. On the other hand, DF-1 cells cultured in 35 mm dishes were co-transfected with pCMV-HA-M and the indicated plasmids. At 24 h post-infection or post-transfection, cells were washed and lysed with immunoprecipitation buffer. After centrifugation, the supernatants were collected and incubated with the corresponding antibodies overnight at 4°C. The immune complexes were recovered by adsorption to protein A+G-Sepharose (Sigma) for 3 h at 4°C. After three washes in immunoprecipitation buffer, the immunoprecipitates were detected by Western blotting.

For pull-down assays, the His-importin β1 or His-importin β1(△336–433) or His-importin α5 fusion protein was expressed in *E. coli* BL21 (DE3) (4 h induction with 1.0 mM IPTG at 30°C), and the soluble His-tagged proteins were purified on Ni-NTA His Bind Resin. GST-M and GST-M/NLSm (4 h induction with 0.5 mM IPTG at 28°C), and GST-importin β1 (4 h induction with 1.0 mM IPTG at 30°C) were expressed in *E. coli* BL21 (DE3), respectively, and the soluble fusion proteins were purified on Glutathione-Sepharose beads. In the GST pull-down experiments, the purified GST-M or GST-M/NLSm or GST-importin β1 protein was immobilized on Gluthatione-Sepharose beads (3 μg protein/10 μL beads). After washing with transport buffer, the immobilized proteins were incubated with the purified His-importin β1 or His-importin β1(△336–433) or His-importin α5 (3 μg for each protein, total volume 40 μL) for 2 h at 4°C. The beads were then washed three times with transport buffer and the bound proteins were eluted from the beads and used for SDS-PAGE followed by Coomassie blue staining or Western blot analysis. In the His pull-down experiments, His*Bind Resin-binded His-importin β1 or His-importin β1(△336-433) (3 μg protein/10 μL resins) was incubated with purified GST-M (3 μg) for 2 h at 4°C. The resins were dealt with as described above, and the target protein GST-M was detected by Western blotting.

### In vitro nuclear import assays

*In vitro* nuclear import assays in digitonin-permeabilized cells were performed as previously described [80]. Briefly, subconfluent HeLa cells were cultured on poly-L-lysine-coated glass coverslips for 1 day and then permeabilized with 70 μg of digitonin/ml for 5 min on ice. The digitonin-permeabilized HeLa cells were rinsed twice with transport buffer and then incubated for 15 min at room temperature with the import mixture. Import reactions contained an energy regenerating system (0.5 mM GTP, 5 mM phosphocreatine, and 0.4 U of creatine phosphokinase), plus various transport factors (0.5 μM importin β1; 3 μM RanGTP; 3 μM RanGDP; 3 μM RanQ69LGTP), plus the GST-M-GFP fusion protein (0.5 μM). The final reaction volume was adjusted to 20 μL with transport buffer. After incubation, the cells were washed with transport buffer and fixed with 3.7% formaldehyde on ice followed by methanol for 5 min at -20°C. After three washes with transport buffer, the nuclei were identified by DAPI staining. The results of GST-M-GFP nuclear import were analyzed with a Nikon fluorescence microscope.

### Virus rescue and pathogenicity assay

For the virus rescue, BSR-T7/5 cells were grown in DMEM medium containing 10% FBS and 1 mg/ml geneticin G418 for five generation before transfection. Cells at 70% confluence in 35 mm dishes were transfected with the full-length cDNA clone (pNDV/SS1GFP or pNDV/SS1GFP-M/NLSm) together with three SS1-derived helper plasmids at a total of 3 μg [77]. At 60 h post-transfection, the cell monolayers and culture supernatants were harvested and inoculated into the allantoic cavities of 10-day-old embryonated SPF chicken eggs. The HA test and DNA sequencing were performed to identify the rescued viruses rSS1GFP and rSS1GFP-M/NLSm. Plaque formation assays and viral titers were performed using standard methods [81]. The pathogenicity assay of the rescued viruses was determined using the standard pathogenicity tests: the MDT test in 10-day-old SPF chicken eggs, and the ICPI test in 1-day-old SPF chicks [81].

### Pathogenicity assessment of the rescued viruses in 4-week-old chickens

The pathogenicity assessment of the rescued viruses rSS1GFP and rSS1GFP-M/NLSm was determined in 4-week-old SPF chickens. Forty-eight chickens were assigned randomly into three experimental groups, consisting of rSS1GFP- (n = 16), rSS1GFP-M/NLSm- (n = 16) and mock- (PBS, n = 16) infected groups. For each group, 6 birds and 10 birds were used for sampling and clinical observation, respectively. Chickens were inoculated via the eye drop/intranasal route with each virus at a dose of 10^5.0^ EID_50_/100 μL per bird, or with 100 μL PBS as the negative control. The birds were monitored for clinical signs daily for 10 dpi. Three birds were euthanized daily from 4–5 dpi for gross lesion observation, and samples of the heart, liver, spleen, lung, kidney, brain, trachea, duodenum, bursa of Fabricius and thymus were collected to detect virus titration in DF-1 cells at 5 dpi. The virus titers were determined as the TCID_50_ per gram (log_10_TCID_50_ g^−1^ tissue) using the endpoint method of Reed and Muench. The part of collected lymphoid tissues (spleen, thymus and bursa of Fabricius) was fixed in 10% neutral formalin, routinely sectioned and stained with hematoxylin-eosin, and then examined for lesions using Nikon light microscopy.

### Immunofluorescence antibody assay

DF-1 cells grown in 12-well plates were treated with the drug ivermectin or DMSO as described previously [82]. Cells were then infected with NDV strain rSS1GFP at an MOI of 0.1 and prepared for immunofluorescence analysis at 12 hpi. At the stipulated time, cells were rinsed with PBS, fixed with 4% paraformaldehyde for 20 min, and then permeabilized with 0.2% Triton X-100 in PBS for 5 min. Cells were rinsed with PBS and blocked with 10% FBS in PBS for 30 min, and then incubated with anti-M polyclonal antibodies diluted in PBS containing 10% FBS for 1 h [16]. After three washes with PBS, the cells were incubated with Alexa Fluor 488 goat anti-rabbit immunoglobulin G antibody (Invitrogen) for 1 h. Cells were counterstained with DAPI to detect nuclei. For siRNA experiments, siRNA-transfected DF-1 cells were infected with rSS1GFP at an MOI of 1, and cells were collected at 6 hpi or 12 hpi to perform immunofluorescence antibody assay as described above. Images were captured with a fluorescence microscope and processed with Adobe Photoshop 7.0 software.

### siRNA treatment and virus infection

The sequences of three pairs of siRNA designed to knockdown importin β1 or importin α5 in DF-1 cells were shown in supplemental Table S1. Negative siRNA control (Cat. No.12935-400) and siRNA transfection reagent were purchased from Invitrogen. For transfection with the siRNA against importin β1 or importin α5, low-passage DF-1 cells were transfected with the indicated siRNAs at a confluence of 80% on 35 mm dishes, and the knockdown efficiency was checked by immunoblot analysis at 48 h post-transfection. To study the effect of importin β1 or importin α5 knockdown on the subcellular localization of M protein and the replication of NDV, the NDV strain rSS1GFP was used to infect importin β1 or importin α5 siRNA-treated DF-1 cells at an MOI of 1. The subcellular localization of M protein was examined by immunofluorescence assay and immunoblotting analysis and at 6 hpi and 12 hpi, respectively. In addition, the cell culture supernatants and cell pellets were collected at the indicated time points (6, 12, 24, 48, and 72 hpi), and the virus titers were determined as 50% tissue culture infective dose (TCID_50_) in DF-1 cells [78]. Moreover, the CPE and green fluorescence in virus-infected cells were observed under fluorescence microscope and the GFP expression level was detected by Western blotting at 24 hpi.

### RNA isolation and qRT-PCR analysis

Total RNA was prepared from siRNA-treated DF-1 cells using Qiagen RNeasy kit according to the manufacturer's protocol. One microgram of total RNA was used for cDNA synthesis by reverse transcription kit (TaKaRa). For quantification of the mRNA of NDV M gene, a SYBR green-based real-time PCR method (TaKaRa) was used, and GAPDH mRNA was quantified to normalize the total RNA concentration between different samples. The primers for detecting the M gene and GAPDH gene were designed with reference to previous publication [16]. The real-time PCR operation was carried out according to the previously described method [78]. The standard cure method was used to analyze the fold change of M gene mRNA expression level. For cell pellets analysis, the NDV M gene expression levels were calculated relatively to the expression of the GAPDH gene. The relative fold change of M gene expression in the cell pellets was calculated as follows: (mRNA expressions of M gene/GAPDH in Control RNAi or Normal cells)/ (mRNA expressions of M gene/GAPDH in importin β1 or importin α5 RNAi cells). The relative fold change of M gene expression in the cell supernatants was calculated as follows: (mRNA expressions of M gene in Control RNAi or Normal cell culture)/ (mRNA expressions of M gene in importin β1 or importin α5 RNAi cell culture).

### Statistical analysis

Statistical analysis was performed using the GraphPad Prism 6.0 software (GraphPad Software Inc., La Jolla, CA, USA). The *p*-values between identified samples were generated using unpaired two-tailed Student's *t*-test. All experiments were repeated at least three times and the results were presented as the mean ± standard deviation (SD). The significance levels were defined as *P < 0.05*.

## Supplementary Material

Supplementary_Material.docx

## Abbreviations

NDV: Newcastle disease virus
HRSV: human respiratory syncytial virus
VSV: vesicular stomatitis virus
MeV: measles virus
SeV: Sendai virus
NiV: Nipah virus
SPF: specific pathogen free
hpi: hours post-infection
dpi: days post-infection
CPE: cytopathic effect
EID_50_: 50% egg infectious dose
TCID_50_: 50% tissue culture infective dose
NLS: nuclear localization signal
NoLS: nucleolar localization signal
NES: nuclear export signal
NPC: nuclear pore complex
RanGDP: GDP-bound small GTPase Ran
RanGTP: GTP-bound small GTPase Ran
