# Supplementary material for: Importin α5 negatively regulates importin β1-mediated nuclear import of Newcastle disease virus matrix protein and viral replication and pathogenicity in chicken fibroblasts
Source: Virulence. 2018 Apr 24;9(1):783–803. doi: 10.1080/21505594.2018.1449507 (PMC5955436; doi:10.1080/21505594.2018.1449507)

**Table S1.** The information of chicken importin β1 and importin α5 siRNA.

| Gene | Name | Sequence(5'→3') | |
| --- | --- | --- | --- |
| importin β1 | RNAi#1 | Sense | GACCUGCUGUGAAGAUGACAUUGUU |
|  |  | Anti-sense | AACAAUGUCAUCUUCACAGCAGGUC |
|  | RNAi#2 | Sense | UAGUCUUGCUGAAGCUGCCUAUGAA |
|  |  | Anti-sense | UUCAUAGGCAGCUUCAGCAAGACUA |
|  | RNAi#3 | Sense | AUGCAGAGCUCCUCAAGUUAUUCUG |
|  |  | Anti-sense | CAGAAUAACUUGAGGAGCUCUGCAU |
| importin α5 | RNAi#1 | Sense | UUUCCAUGUUGUUCAUUUGAGCCUC |
|  |  | Anti-sense | GAGGCUCAAAUGAACAACAUGGAAA |
|  | RNAi#2 | Sense | CAGAAAUUCCGAAAGCUCCUUUCUA |
|  |  | Anti-sense | UAGAAAGGAGCUUUCGGAAUUCUG |
|  | RNAi#3 | Sense | CAGAGACUAUGUACUGGACUGUAAU |
|  |  | Anti-sense | AUUACAGUCCAGUACAUAGUCUCUG |
| Control | Negative RNAi | Sense | UUCUCCGAACGUGUCACGUTT |
|  |  | Anti-sense | ACGUGACACGUUCGGAGAATT |

**Figure S1.** Nucleotide sequence analysis of the mutations introduced in the NLS motif of the M gene. The rescued virus was plaque purified and passaged five times in 10-day-old SPF chicken eggs, and the nucleotide replacements introduced in the M gene were indicated.

**
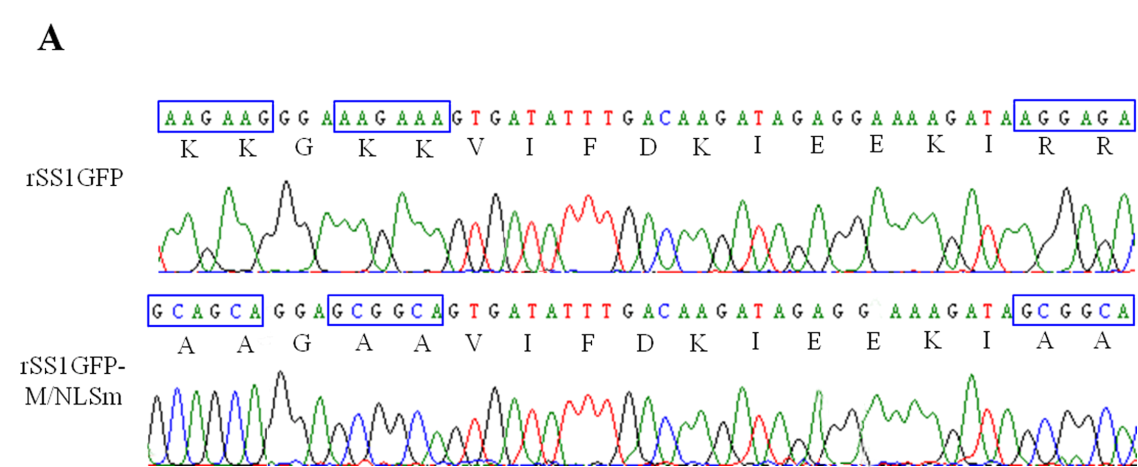
**

**Figure S2.** Construction of the NDV full-length cDNA clone carrying NLS mutation in the M protein


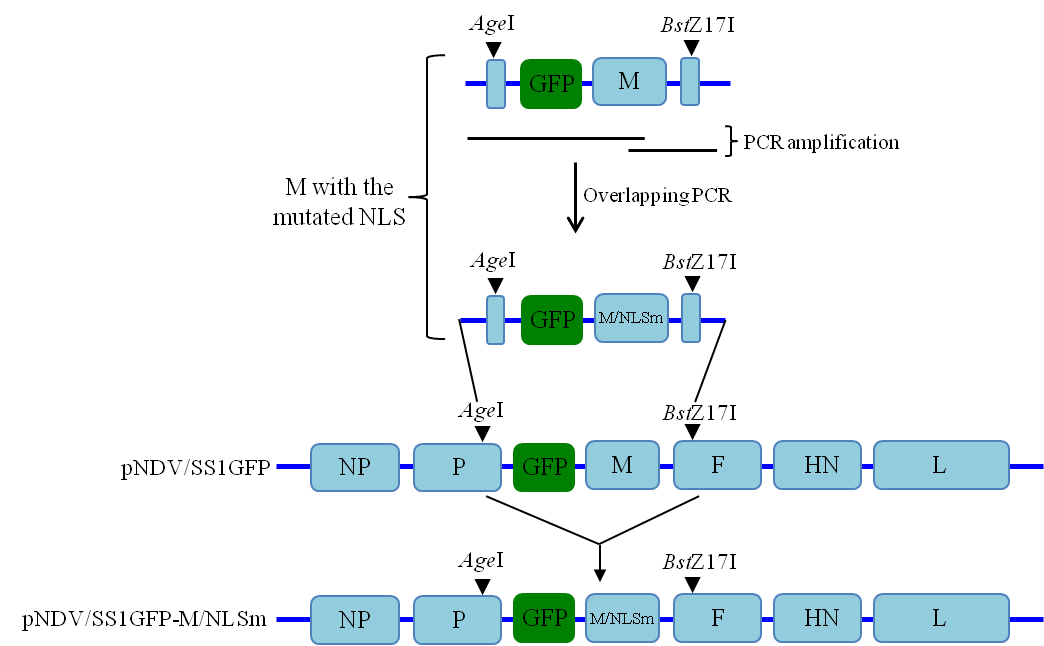

Supplement: Supplementary_Material.docx [file kvir-09-01-1449507-s001.docx]
